# Hemophagocytic lymphohistiocytosis as an etiology of bone marrow failure

**DOI:** 10.3389/fonc.2022.1016318

**Published:** 2022-10-27

**Authors:** Jonathan Paolino, Nancy Berliner, Barbara Degar

**Affiliations:** ^1^ Department of Pediatric Oncology, Dana-Farber Cancer Institute, Harvard Medical School, Boston, MA, United States; ^2^ Division of Hematology, Brigham and Women’s Hospital, Harvard Medical School, Boston, MA, United States

**Keywords:** hemophagocytic lymphohistiocytosis, hypercytokinaemia, familial hemophagocytic lymphohistiocytosis (FHL), macrophage activation syndrome (MAS), cytopenia, bone marrow failure (BMF)

## Abstract

Hemophagocytic lymphohistiocytosis (HLH) is a syndrome of multiorgan system dysfunction that is caused by hypercytokinemia and persistent activation of cytotoxic T lymphocytes and macrophages. A nearly ubiquitous finding and a diagnostic criterion of HLH is the presence of cytopenias in ≥ 2 cell lines. The mechanism of cytopenias in HLH is multifactorial but appears to be predominantly driven by suppression of hematopoiesis by pro-inflammatory cytokines and, to some extent, by consumptive hemophagocytosis. Recognition of cytopenias as a manifestation of HLH is an important consideration for patients with bone marrow failure of unclear etiology.

## Introduction

Hemophagocytic lymphohistiocytosis (HLH) is a rare syndrome in which immune dysregulation and severe pathologic inflammation result in multiorgan dysfunction. While affected patients share certain clinical features, the inciting event, nature of external predisposing factors, and presence of underlying genetic predisposition vary widely. In HLH, the inability of natural killer (NK) and CD8+ cytotoxic T lymphocytes (CTLs) to provide critical negative feedback in response to an immunologic trigger leads to uncontrolled activation of CTLs and macrophages and initiation of a “cytokine storm” (1–3). Peripheral blood cytopenias are a universal feature of HLH and are an important diagnostic criterion for the syndrome (4–6). The causes of bone marrow failure in HLH are multifactorial, heterogeneous, and incompletely understood.

Since it was first described in 1939 by Scott and Robb-Smith, HLH has remained diagnostically challenging with highly variable clinical presentation and no single pathognomonic feature (7–10). Historically, HLH has been dichotomized as “primary” (familial or FHL) for those with a family history of HLH or predisposing genetic mutation, or “secondary” for those without an identified mutation but with an underlying infectious, rheumatologic, or malignant disease (6, 11). There is, however, considerable overlap between the two groups, with increasing understanding of the role of novel mutations and monoallelic variants in the pathogenesis of HLH (12–14). In addition, FHL may be initiated by an inciting infectious or inflammatory insult, further blurring the line between “primary” and “secondary” groups.

The constellation of clinical findings and laboratory abnormalities observed in HLH reflect the common pathway through which HLH progresses. The Histiocyte Society developed standard diagnostic criteria for the disorder and undertook the HLH94 clinical trial with the goal of improving survival of children with familial HLH, a uniformly fatal diagnosis at that time (**Table 1**) (17). However, the suitability of these criteria for diagnosing various forms of secondary HLH is not established. Modified criteria for patients with rheumatologic disease and underlying malignancy have been proposed (16, 18, 19). A scoring system termed the Optimized HLH inflammatory (OHI) index was recently developed to aid in the diagnosis and management of HLH in the context of hematologic malignancy (20).

**Table 1:** Diagnostic criteria for HLH.

	HLH 2004 (4, 6, 15)	H-Score (16)	
Category:	Criteria: (5 of the following 8)	Criteria:	Points
*Clinical*			
Fever	Temperature ≥ 38.3°C	Temperature <38.4°C	0
		Temperature 38.4–39.4°C	33
		Temperature >39.4°C	49
Organomegaly	Splenomegaly	None	0
		Hepatomegaly OR splenomegaly	23
		Hepatomegaly AND splenomegaly	38
*Pathological*			
Hemophagocytosis	Bone marrow, spleen, or lymph nodes	None	0
		Bone marrow	35
*Laboratory*			
Cytopenias	≥ 2 lineages in peripheral blood:	1 lineage	0
	Hemoglobin < 9 g/dL Platelets < 100 × 10^3^/μL Neutrophils < 1 × 10^3^/μL	2 lineages	24
		3 lineages	34
Hypertriglyceridemia	Fasting triglycerides ≥ 265 mg/dL	< 1.5 mmol/L	0
		1.5–4 mmol/L	44
	**AND/OR**	> 4 mmol/L	64
Hyperfibrinogenemia	Fibrinogen ≤ 150 mg/dL	> 250 mg/dL	0
		≤ 250 mg/dL	30
Ferritin	≥ 500 ng/mL	< 2,000 ng/mL	0
		2,000–6,000 ng/mL	35
		> 6,000 ng/mL	50
Soluble CD25 (IL-2 receptor)	≥ 2,400 U/mL	N/A	
NK cell activity	Low or absent NK-cell activity	N/A	
SGOT/AST	N/A	< 30 IU/L	0
		≥ 30 IU/L	19
	**OR:**		
*Predisposition*	Known pathogenic mutation of PRF1, UNC13D, STXBP2, Rab27a, STX11, SH2D1A, or XIAP	No immunosuppression	0
		Long term immunosuppression	18

NA, Not applicable.

### Peripheral blood findings

Cytopenias affecting at least two cell lines are a cardinal feature of HLH. Thrombocytopenia is almost always present (4, 21). The platelet count may initially be normal or modestly depressed; however, it often falls as the disease progresses (22). Normocytic anemia with reticulocytopenia is also common. Leukopenia and neutropenia are more variably present (22). For example, 92% of children enrolled on the HLH2004 trial demonstrated bi-cytopenia (4). Similarly, among 775 adult patients with HLH, significant cytopenias were present in the majority: platelets < 100 × 10^3^/μL in 78% and < 10 × 10^3^/μL in 6%; hemoglobin of < 9 g/dL in 67% and < 7 g/dL in 22%; absolute neutrophil count (ANC) < 1 × 10^3^/μL in 42% and < 0.5 × 10^3^/μL in 23% (21).

### Bone marrow findings

**Figure 1:**
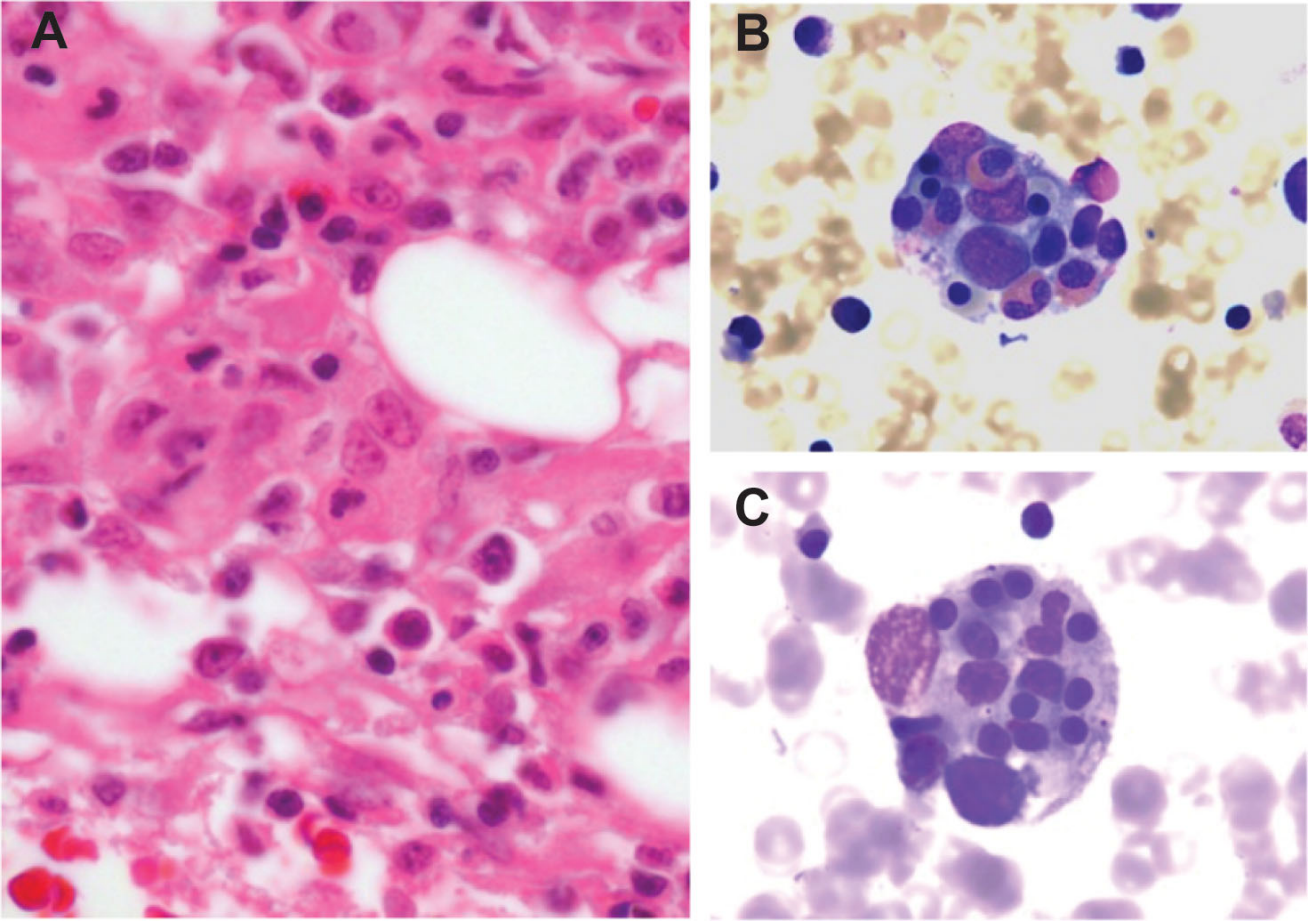
Histopathology of HLH **(A)** Histopathology of a bone marrow section in HLH demonstrates marrow infiltration by macrophages (hematoxylin and eosin stained, 400x magnification). **(B, C)** Bone marrow aspirate stain demonstrates macrophages engulfing hematopoietic cells, including eosinophils and erythroid precursors (Wright-Giemsa stained, 1000x magnification).

In patients with HLH, the bone marrow demonstrates diffuse histiocytic infiltration, histiocyte hyperplasia, and variable numbers of cytotoxic T-cells (9, 22). Hemophagocytosis, the pathologic finding of activated macrophages engulfing erythrocytes, leukocytes, platelets, and their precursor cells, is variably present (**Figure 1**). This process occurs not just in the bone marrow but throughout the reticuloendothelial system, including in the spleen, liver, and lymph nodes. While the finding of hemophagocytosis in bone marrow or tissue supports the diagnosis of HLH in the proper clinical context, it is neither essential for the diagnosis nor pathognomonic. In a series of 122 children with HLH from the Histiocyte Society’s International Registry, only 75% had evidence of hemophagocytosis at diagnosis (23). Conversely, hemophagocytosis may be present in patients without HLH. In one study, among 107 adult patients who died of multiorgan failure unrelated to HLH, none of whom fulfilled standard HLH diagnostic criteria, 69 (64.5%) demonstrated histiocytic hyperplasia and hemophagocytosis on postmortem bone marrow analysis (24). Because of its non-specific nature, hemophagocytosis must be carefully considered in the context of other clinical findings.

### Familial HLH

**Figure 2:**
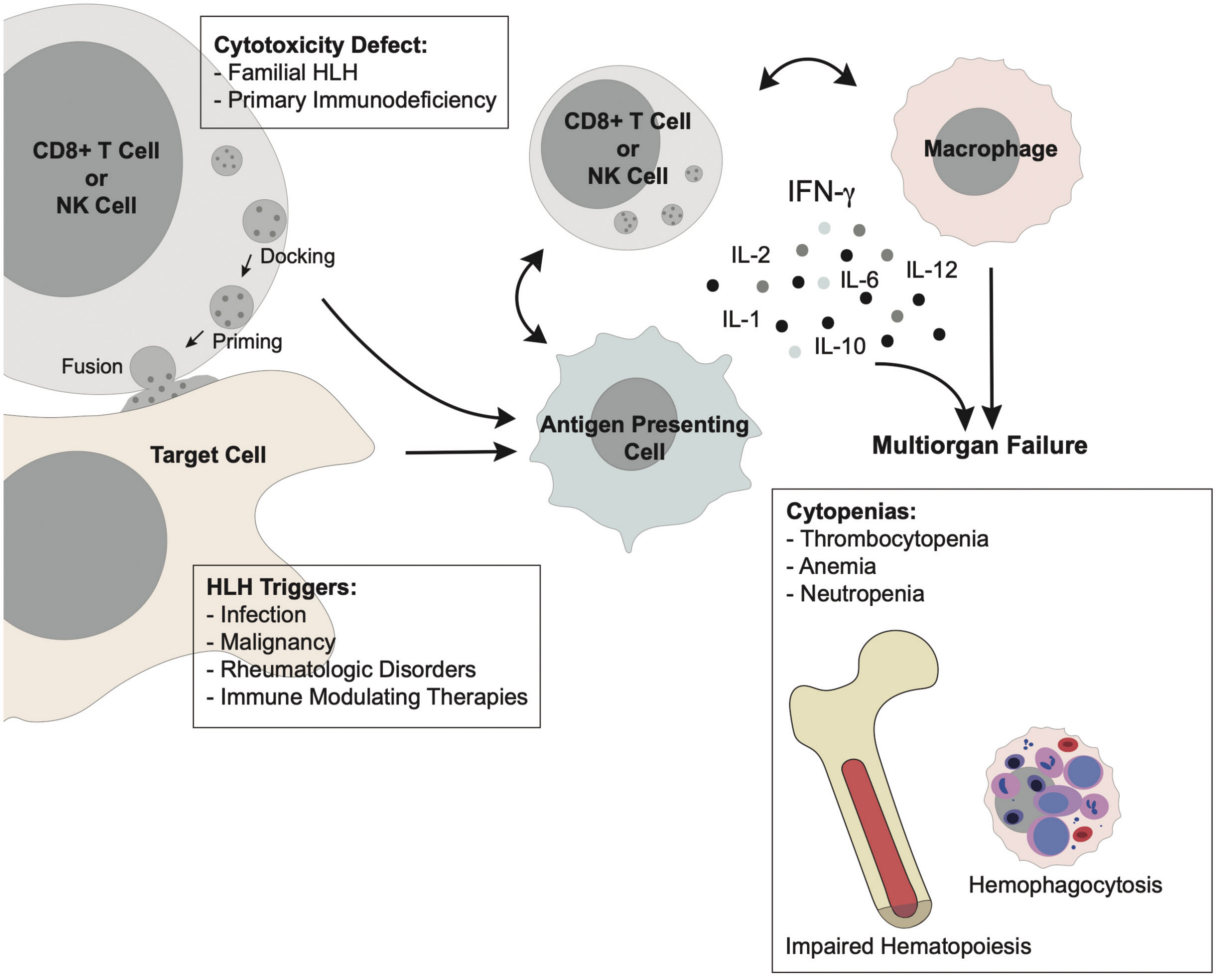
Pathophysiology of HLH Primary HLH arises from defective cytotoxicity in CD8+ T cells or NK cells, allowing persistence of an antigenic stimulus. Secondary HLH arises from unchecked immune activation secondary to infection, malignancy, rheumatologic disorders, or immune-modulating therapy. This immune activation acts through a common pathway in which uncontrolled stimulation of CD8+ T cells and macrophages results in hypercytokinemia which drives the clinical manifestations of multi-organ failure. Cytopenias in the form of neutropenia, anemia, and thrombocytopenia are ubiquitous in HLH downstream of excessive production of IFN- γ and other inflammatory cytokines. This “cytokine storm” suppresses normal hematopoiesis and results in diffuse hemophagocytosis by activated macrophages.

Genetic linkage studies performed on families with HLH led to the discovery of bi-allelic *Prf1* mutations as the cause of FHL-2 in 1999 (25). Perforin, encoded by *Prf1*, is a constituent of cytotoxic granules within CD8+ cytotoxic T cells and NK cells (26). Activated CTLs and NK cells form an immunologic synapse with target cells, such as virally infected or cancer cells, allowing cytotoxic granules to undergo a complex series of events through which they dock, prime, and fuse with the cytoplasmic membrane to release their contents (**Figure 2**) (27–31). This leads to perforin-dependent pore formation in the target cell membrane and allows serine protease granzymes to induce apoptosis of the target cell (26, 32, 33). Absent or reduced expression of functional perforin impairs effector function and clearance of the inflammatory insult, resulting in persistent activation of this pathway (25).

The finding of *Prf1* mutations in families with FHL-2 was followed by the discovery of the genetic basis for FHL-3, FHL-4, and FHL-5, which are caused by mutations affecting expression or function of Munc13-4, Syntaxin11, and Munc18-2, respectively (**Table 2**) (34–36). These proteins are necessary for docking, priming, and fusion of cytotoxic granules with the T or NK cell membrane (34–36). Other genetic syndromes associated with defective trafficking of cytotoxic granules, including Chediak-Higashi syndrome, Griscelli syndrome type 2, Hermansky-Pudlak syndrome type 2, and RhoG deficiency, are also associated with HLH (**Table 2**) (1, 37–40, 46).

**Table 2:** Genetic mutations associated with primary HLH.

HLH type or syndrome	Gene	Protein	Affected function
**Mutations affecting degranulation**			
Familial HLH type 3 (34)	*UNC13D*	Munc13-4	Vesicle priming
Familial HLH type 4 (35)	*STX11*	Syntaxin11	Vesicle fusion
Familial HLH type 5 (36)	*STXBP2*	Munc18-2	Vesicle fusion
Chediak-Higashi syndrome (37)	*LYST*	LYST	Vesicle trafficking
Griscelli syndrome type 2 (38)	*RAB27A*	RAB27A	Vesicle docking
Hermansky-Pudlak syndrome type 2 (39)	*AP3B1*	AP-3	Vesicle trafficking
RhoG deficiency (40)	*RHOG*	RhoG	Vesicle docking
**Mutations affecting pore formation in target cells**			
Familial HLH type 2 (25)	*PRF1*	Perforin	Pore formation
**Mutations affecting CTL or NK development, survival, and/or regulation**			
X-linked lymphoproliferation type 1 (41, 42)	*SH2D1A*	SAP	CTL and NK signaling
X-linked lymphoproliferation type 2 (43)	*BIRC4*	XIAP	CTL and NK apoptosis
**Mutations affecting inflammasome regulation**			
NLRC4 Inflammasomopathies (44, 45)	*NLRC4*	NLRC4	Inflammasome regulation

Signaling lymphocytic activation molecule-associated protein (SAP), X-linked inhibitor of apoptosis (XIAP), NLR family, CARD domain-containing protein 4 (NLRC4).

X-linked lymphoproliferative disease type 1 (XLP1) is caused by hemizygous mutations in signaling lymphocytic activation molecule-associated protein (SAP) (41, 42). SAP plays a critical role in T cell response through interactions with signaling lymphocyte activation molecules (SLAM) family receptors (47, 48). Patients with XLP1 develop severe, overwhelming immune dysregulation in the setting of Epstein-Barr virus (EBV) infection (47, 48). In X-linked lymphoproliferative disease type 2 (XLP2), mutations in X-linked inhibitor of apoptosis (XIAP) lead to reduced survival of T and NK cells (43, 49). Patients with XIAP deficiency have a high incidence of HLH (43, 49). Collectively, these disorders result in ineffective cytotoxicity of CTLs and NK cells which results in persistence of the antigenic stimulus and predisposes to the pathogenesis of HLH.

In patients with HLH, uncontrolled activation of immune cells leads to production of pro-inflammatory cytokines, which in turn act to amplify immune dysregulation in an unchecked positive feedback loop. Multiple cytokines have been implicated in the pathogenesis of HLH, including interferon-γ (IFN- γ), interleukin (IL)-1, IL-2, IL-6, IL-12, IL-18, and tumor necrosis factor-alpha (TNF-α) (50–52). In FHL, interferon-γ has emerged as a key driver of disease activity (2, 53–55). Importantly, further insight into the role of individual cytokines in HLH has potential therapeutic implications (51, 56, 57). For example, neutralization of IFN-γ with emapalumab led to its approval for treatment of FHL in patients with refractory HLH or intolerance to standard chemotherapy (55).

### Secondary HLH

Unlike FHL, which typically presents in infancy or early childhood, secondary HLH is far more prevalent in the older child and adult population. Secondary HLH arises in the setting of an immune stimulus associated with a malignancy, infection, rheumatologic disorder, primary immunodeficiency syndrome, and/or immune-modulating treatment, as outlined in **Table 3** (1, 6, 58). The two most common triggers of secondary HLH are infection and malignancy (1, 21, 66–68). Epstein-Barr virus and other herpes viruses are especially frequent; in one study, herpes viruses were identified in 62% of virus-associated HLH cases in adults (21, 59). Lymphoma and leukemia underlie the vast majority of cases of malignancy-associated HLH (19, 58, 69). In addition, several newly developed cancer therapies, such as immune checkpoint inhibitors, monoclonal antibodies, and chimeric antigen receptor T-cells, may also lead to hyperinflammation and/or cytokine release syndrome that may resemble HLH (58).

**Table 3:** Secondary HLH.

Etiology (1, 6)	Example disorders or therapies
HLH in the context of malignancy (19, 58)	Lymphomas, acute myeloid leukemias, acute lymphoblastic leukemias, lung cancers, colon cancers
HLH in the context of infection (59–61)	Epstein-Barr virus (EBV), cytomegalovirus, herpes simplex virus (HSV), human immunodeficiency virus (HIV), COVID-19
HLH in the context of a rheumatologic disorder (19, 62, 63) (Macrophage activation syndrome)	Kawasaki disease, systemic lupus erythematosus (SLE), systemic juvenile idiopathic arthritis (SJIA), rheumatoid arthritis (RA)
HLH in the context of primary immune deficiency (6, 64)	Severe combined immunodeficiency (SCID), chronic granulomatous disease (CGD), X-linked immunodeficiency with magnesium defect (XMEN)
Immune-effector-related hyperinflammatory syndromes (58, 65)	Immune checkpoint inhibitors, chimeric antigen receptor (CAR) T-cell therapy, hematopoietic stem cell transplant, solid organ transplant

Patterns of T cell activation differ between primary and secondary HLH, reflecting potential differences in the underlying pathogenesis (70). In one study of T cell activation patterns, patients with primary or virus-associated secondary HLH were found to have significantly higher expression of HLA-DR in CD8 + T cells, a marker of T-cell activation, when compared to those with secondary HLH without a viral trigger (64.4% and 61.5% vs. 21%, respectively) (70). Cytokine profiles are frequently similar but may vary based on the underlying inflammatory trigger (51, 71). IFN- γ, TNF-α, IL-10, and IL-18 are commonly elevated in secondary HLH (72–75). Despite the etiologic heterogeneity of secondary HLH, the pathophysiology follows a similar common pathway in which persistent antigenic stimulation leads to an amplified and unchecked immune response, excessive cytokine production, and the resultant development of hallmark features of HLH (1, 50, 57).

### Cytopenias in HLH

Many factors contribute to the development of cytopenias in patients with HLH, including impairment of hematopoiesis mediated through the action of pro-inflammatory cytokines, consumption of hematopoietic progenitors due to hemophagocytosis throughout the reticuloendothelial system, shortened survival of blood cells due to hepatosplenomegaly and/or disseminated intravascular coagulopathy, co-existing viral infection, marrow invasion by cancer, and treatment-related myelosuppression (21, 51, 76–79). Despite the complex interactions of these factors, two distinct drivers are attributable to the phenomenon of HLH itself: impaired hematopoiesis as a result of hypercytokinemia and consumptive hemophagocytosis by activated macrophages.

#### Impaired hematopoiesis

Suppression of hematopoiesis by interferon-γ and other inflammatory cytokines is well described (79–82). Indeed, IFN-γ has been implicated in the pathogenesis of aplastic anemia (83, 84). In the setting of inflammation, interferon-γ exhibits both stimulating and suppressive effects on hematopoietic precursors in a lineage-dependent manner (80, 85). IFN-γ plays a key role in myelopoiesis, directing differentiation to either monocyte or neutrophil populations (86). In contrast, IFN-γ exhibits a predominantly suppressive effect on hematopoietic erythroid progenitors and disrupts thrombopoietin signaling in hematopoietic stem cell precursors (87–89). Interestingly, interferon-γ and TNF-α, two cytokines frequently elevated in patients with HLH, have demonstrated the potential to synergistically suppress bone marrow erythroid and multipotential progenitor cells (79). The role of inflammatory cytokines as an etiology of bone marrow failure has substantial implications in the context of the hypercytokinemia observed in patients with HLH.

*In vivo* preclinical models of HLH support the role of IFN-γ in the development of impaired hematopoiesis. In one study, IFN-γ knockout abrogated the development of anemia in a murine model of toll-like receptor 9 (TLR9)-induced fulminant macrophage activating syndrome (90). Interestingly, in this study, both IFN-γ wild-type and knockout mice developed an MAS/HLH-like syndrome following exposure to a TLR9 agonist, suggesting that the clinical phenotype was not mediated by IFN-γ alone. The IFN- γ knockout mice, however, did not develop anemia despite the presence of hemophagocytosis. These mice were found to have compensatory splenic erythroid precursor production, leading authors to conclude that dyserythropoiesis, not hemophagocytosis, was primarily responsible for anemia in this model (90). Further supporting the role of interferon-γ in the development of cytopenias are the findings that anti-interferon-γ antibodies correct peripheral blood cytopenias and histiocytic infiltration of the marrow, liver, and spleen in perforin and Rab27a-deficient mice (2, 53).

#### Consumptive hemophagocytosis

Hemophagocytosis is frequently observed in HLH, although it is neither specific nor required for the diagnosis. The role of hemophagocytosis in the development of pancytopenia in HLH is uncertain. In one animal model, sustained exposure to IFN- γ in wild-type mice induced the development of dose-dependent normocytic anemia and compensatory reticulocytosis (54). In this model, mice were infused with IFN- γ over a five-day period, during which anemia became apparent within 48 hours. There was no change in red blood cell morphology or evidence of a significant hemolytic process (54). Anemia was accompanied by thrombocytopenia and leukopenia, and was associated with diffuse hemophagocytosis. The temporal relationship between the start of IFN- γ infusion and the development of anemia, as well as the accompanying reticulocytosis, led the authors to conclude that the cytopenias observed in this model were likely to be predominantly the result of a consumptive process secondary to acute inflammation rather than suppression of hematopoiesis (54). These findings further implicate IFN- γ as a key driver of the cytopenias observed in patients with HLH.

## Conclusions

Hemophagocytic lymphohistiocytosis (HLH) is a hyperinflammatory syndrome that results from persistent activation of cytotoxic T lymphocytes and macrophages. The underlying causes of HLH are heterogeneous; however, peripheral blood cytopenias are almost universally present. The mechanism of cytopenias is multifactorial and may be exacerbated by concomitant effects by factors such as infection of hematopoietic progenitors, bone marrow infiltration, and myelosuppressive therapy. Inflammatory cytokines, especially interferon-γ, play a significant role in suppressing hematopoiesis in HLH, leading to cytopenias in animal models and patients. To a variable extent, consumptive hemophagocytosis throughout the reticuloendothelial system by macrophages contributes. Importantly, recognition of cytopenias as a manifestation of HLH is essential for patients with bone marrow failure of unclear etiology.

## Author contributions

The manuscript was written by JP and extensively edited by NB and BD. All authors contributed to the article and approved the submitted version.

## Funding

JP was supported by NIH 5T32HL007574-38.

## Conflict of interest

The authors declare that the research was conducted in the absence of any commercial or financial relationships that could be construed as a potential conflict of interest.

## Publisher’s note

All claims expressed in this article are solely those of the authors and do not necessarily represent those of their affiliated organizations, or those of the publisher, the editors and the reviewers. Any product that may be evaluated in this article, or claim that may be made by its manufacturer, is not guaranteed or endorsed by the publisher.
